# Radiorecovery Activity of Dicopper(II) Tetrakis(3,5-Diiso propylsalicylate) Includes Recovery of Radiation-Induced Loss of Body Mass and Impaired Mouse Locomotion

**DOI:** 10.1155/MBD.1999.121

**Published:** 1999

**Authors:** Renada D. Henderson, Timothy D. Henderson, Henry J. Irving, John R. J. Sorenson

**Affiliations:** 1 Department of Medicinal Chemistry College of Pharmacy University of Arkansas for Medical Sciences Campus Little Rock Arkansas 72205 USA

## Abstract

Dicopper(II) tetrakis(3,5-diisopropylsalicylate), [Cu(II)_2_(3,5-DIPS)_4_], is effective in increasing survival of lethally irradiated mice when it is administered *after* irradiation. The possibility that this radiorecovery activity might also facilitate recovery from radiation-induced impaired increase in body mass and locomotion was examined. Cu(II)_2_(3,5-DIPS)_4_ was used to treat LD 50/30 gamma irradiated female C57BL/6 mice *after* irradiation. A dose of 0, 5, 10, or 20 μmol Cu(II)_2_ (3, 5-DIPS)_4_ /kilogram of body mass was administered subcutaneously 3 hrs after LD 50/30 irradiation and change in body mass and locomotor activity measured daily throughout the 30 day post-irradiation period. Treatment with 5, 10, or 20 μmol Cu(II)_2_ (3,5-DIPS)_4_ /kg of body mass increased survival, which was statistically significant for the 10 μmol /kg of body mass-treated group compared to the vehicle-treated group (P<0.05), significantly (P<0.05) increased recovery of locomotion from days 13 to 15 post-irradiation onward for all treated groups compared to vehicle-treated mice, and increased recovery of body mass gain from day 14 onward for the 20 μmol /kg of body mass-treated group (P<0.001) and day 21, although not statistically significant, for the 10 μmol /kg of body mass-treated group. There were no statistically significant differences between the increase in survival, recovered increase in body mass, and recovered increase in locomotion for mice treated with 10 μmol or 20 μmol Cu(II)_2_(3,5-DIPS)_4_ /kg on day 30 post-irradiation. It is concluded that Cu(II)_2_(3,5-DIPS)_4_ in addition to increasing survival of irradiated mice increases the rate of recovery of radiation-induced decrease in body mass and locomotion.


### RADIORECOVERY ACTIVITY OF DICOPPER(II) TETRAKIS(3,5-DIISOPROPYLSALICYLATE) INCLUDES RECOVERY OF RADIATION-INDUCED LOSS OF BODY MASS AND IMPAIRED MOUSE LOCOMOTION

Renada D. Henderson, Timothy D. Henderson, Henry J. Irving, and John R. J. Sorenson*

Department of Medicinal Chemistry, College of Pharmacy, University of Arkansas for Medical Sciences Campus, Little Rock, Arkansas 72205, USA
E-mail: sorensonjohnr@exchange.uams.edu

Abstract

Dicopper(II) tetrakis(3,5-diisopropylsalicylate), [Cu(II) 2(3,5-DIPS) 4], is effective in increasing survival of lethally irradiated mice when it is administered *after* irradiation. The possibility that this radiorecovery activity might also facilitate recovery from radiation-induced impaired increase in body mass and locomotion was examined. $Cu(II)_2(3,5\text{-}DIPS)_4$ was used to treat $LD_{50/30}$ gamma irradiated female C57BL/6 mice *after* irradiation. A dose of 0, 5, 10, or 20 µmol $Cu(II)_2(3,5\text{-}DIPS)_4$/kilogram of body mass was administered subcutaneously 3 hrs after $LD_{50/30}$ irradiation and change in body mass and locomotor activity measured daily throughout the 30 day post-irradiation period. Treatment with 5, 10, or 20 µmol $Cu(II)_2(3,5\text{-}DIPS)_4$/kg of body mass increased survival, which was statistically significant for the 10 µmol/kg of body mass-treated group compared to the vehicle-treated group ($P<0.05$), significantly ($P<0.05$) increased recovery of locomotion from days 13 to 15 post-irradiation onward for all treated groups compared to vehicle-treated mice, and increased recovery of body mass gain from day 14 onward for the 20 µmol/kg of body mass-treated group ($P<0.001$) and day 21, although not statistically significant, for the 10 µmol/kg of body mass-treated group. There were no statistically significant differences between the increase in survival, recovered increase in body mass, and recovered increase in locomotion for mice treated with 10 µmol or 20 µmol $Cu(II)_2(3,5\text{-}DIPS)_4$/kg on day 30 post-irradiation. It is concluded that $Cu(II)_2(3,5\text{-}DIPS)_4$ in addition to increasing survival of irradiated mice increases the rate of recovery of radiation-induced decrease in body mass and locomotion.

### Introduction

Ionizing radiation causes physical and behavioral decrements in humans. Acute radiation injury produces physical symptoms of fatigue, weakness, nausea, emesis, and diarrhea which considerably alter physical behavior. Limited data regarding cognitive behavioral decrements associated with people exposed to radiation document decreased brain blood flow, a marked increase in anticipatory stress, and reduced performance level causing such problems as impaired completion of imbedded-figures tasks and decreased persistence in solvable behavior tasks when compared with a non-irradiated control groups [1,2]. These tests are used to measure speed and accuracy in decision making of subjects who must reappraise their situation continually and make constant decisions in their attempt to find the correct pathway through a maze as rapidly as possible and with the fewest mistakes. The ability to undertake rescue efforts and understand the behavior decrements of irradiated individuals, which include themselves, following a nuclear accident or radiation exposure of space crews is a matter which will require the attention of those responsible for protection of both the civilian population and astronauts. Understanding that radiation-induced alterations in behavior are likely to interfere with the effective functioning of rescue teams and space mission crews deserves further study [3].

Radiation injury also effects the health and behavior of animals. Mice express symptoms similar to humans including reduced body mass and locomotion [3-7]. Mice exhibit decreased locomotor activity in an open field maze, and perform poorly in shock avoidance and roto-rod tests as a result of radiation exposure [5]. Radiation-induced Performance Decrement (PD) occurring in mice begins with Early Transient Incapacitation (ETI) period and continues for at least 30 days post irradiation [6,7]. Exposure to ionizing radiation has been shown to decrease locomotor activity as much as 40% for an entire 150 day post irradiation period [7].

Several compounds have been tested throughout the entire post-irradiation period for mitigation of PD. Caffeine, a central nervous system stimulant, was shown to reduce PD in mice following irradiation but provided no protection against lethality [8].

Three radioprotectants, WR-1065, 2[(3-aminopropyl)amino]propanethiol, WR-2721, 2[(3-aminopropyl)amino]ethanethiol dihydrogen phosphate ester and WR-3689, 2[(3-methylaminopropyl)amino]-ethanethiol dihydrogen phosphate are effective in increasing survival at doses close to their $LD_{50}$ doses. Their acute toxicities in humans caused them to be abandoned as radioprotectants. The effective radioprotectant dose of these drugs is such that their concomitant acute and behavioral toxicities causes them to be unacceptable for situations where rapid and skilled performance is required [7,9]. An even greater concern is that WR-2721 acting synergistically with the stress of roto-rod performance causes an increase in lethality [6].

During space travel, astronauts will be placed in physically and intellectually demanding situations. Thus, although WR-2721 is an effective protector against lethality, it is not an effective protector against PD [6]. The search then continues for a compound that has two very important activities, protecting against radiation lethality and decreasing radiation-induced behavioral decrements or at the very least not exacerbating them.

It has been shown that post-irradiation treatment with $Cu(II)_2(3,5\text{-DIPS})_4$ increases the rate of recovery of immunocompetency and histopathology and it increases survival of irradiated mice at doses much lower than its $LD_{50}$ dose(10). The $LD_{50}$ dose is 13 times greater than the maximally effective dose of this complex, 20 μmol/kg of body mass, when given to female mice before irradiation(10). Treatment with 2.5, 5, or 10 μmol/kg of body mass $Cu(II)_2(3,5\text{-DIPS})_4$ which are 1/26 to 1/105 the $LD_{50}$ dose in female mice, produced survival of 88%, 92%, or 92% respectively.

Objectives of this study were to determine whether or not $Cu(II)_2(3,5\text{-DIPS})_4$ treatment after irradiation facilitates recovery from radiation-induced behavioral decrements and recovery of lost body mass in a dose-response manner. Results of this study demonstrate that treatment with $Cu(II)_2(3,5\text{-DIPS})_4$ does facilitate recovery of radiation-induced loss of body mass and behavioral (locomotion) decrements in conjunction with an increase in survival in a dose related fashion.

### Methods

Purified 3,5-diisopropylsalicylic acid was purchased from Aldrich Chemical Company. Copper(II) chloride dihydrate purchased from Spectrum Chemical Company was used without further purification. $Cu(II)_2(3,5\text{-DIPS})_4$ [12] was synthesized according to published methods [13]. Polyvinyl alcohol (P-1763) was purchased from Sigma. Propylene glycol (Sigma) was microbiologically purified by Millipore filtration through a 0.2 μm filter. A 1.4% mass/volume polyvinyl alcohol (Sigma) solution was prepared by dissolving 3.5 mg of polyvinyl alcohol in saline at 90°C with stirring, cooling to room temperature, and diluting to 250 ml.

Suspensions of $Cu(II)_2(3,5\text{-DIPS})_4$ were prepared in a Bellco HEPA laminar flow hood (Bellco Technologies, Vineland, New Jersey) by first wetting the complex with 4 grams of propylene glycol, 4% mass/volume of the final suspension, in a glass Potter-Elvejhem homogenizer with a Teflon pestle, and diluting with 1.4% polyvinyl alcohol in saline. The resulting paste was gradually diluted with polyvinyl alcohol in saline with homogenization. This suspension was then slowly diluted to the final volume in a graduated cylinder with the rinse from the homogenizer and mixed by vortex stirring. A volume of 0.5 ml of this suspension contained the 20 μmol/kg of body mass dose of complex. Twelve and one-half milliliters of this suspension was then diluted 1:1 with a solution of 4% propylene glycol and 1.4% polyvinyl alcohol in saline to obtain a suspension containing the 10 μmol/kg of body mass dose in 0.5 ml of suspension. This dilution procedure was repeated to obtain a suspension containing the 10 μmol/kg of body mass dose in 0.5 ml of suspension.

All suspensions were vortex stirred prior to administration and stirred throughout the administration period as a standard procedure. All doses were administered with all-glass syringes to avoid contamination from rubber tips on pistons of disposable syringes by zinc and copper compounds used in molding and to prevent oxidation, respectively, of these tips.

Single-Dose samples (0.5 ml) were placed in metal-free culture tubes prior to initiation of treatment and after treatment of each group. These samples were subsequently analyzed for copper by atomic absorption spectrophotometry using established interference-free methods [14] and diluted Cation Cal (Baxter Health Care Corp., Miami, Florida) as an external standard to determine the actual treatment dose. These determined doses were found to vary within ±5% of the intended dose. This procedure is recommended to provide assurance that the actual dose given is the intended dose.

All animal experiments were approved by the institution's Animal Care and Use Committee which has an Animal Welfare Assurance on file with the Office of Protection from Research Risks.

One hundred C57BL/6 nine-week old 19 to 21 gram female mice acclimatized to 12-h 6:00 am to 6:00 pm light/dark cycle and constant $72^0$ C temperature quarters were randomly assigned to one of 20 Polycarbonate (11.5"L X 7.5"W X 5:H) cages, five per cage, and given food and water *ad libitum*. These mice were ear-punched under a mild anesthesia (Metafane) for identification. Prior to irradiation, individual average locomotion was determined for all mice for two minutes to establish a normal level of activity on consecutive days in the morning in an empty cage placed on an Automex Locomotion Counter (Columbus Instruments, Columbus, Ohio). The Sensitivity Level was set at 100 using 2 inch strip of aluminum foil around the top of the cage. Mice usually have a large variation in activity and having them serve as their own control allowed the determination of change in the morning (locomotion) movement about the cage beginning on the day following irradiation and treatment using the same cage size and activity devices. Body mass was measured daily following the determination of locomotion throughout the experiment.

All mice received a whole body dose of 8 Gy, 1.3 8 Gy/min, from 1100 to 1215 hrs Central Standard time. For irradiation two mice were placed in one of four well-ventilated, Plexiglas containers and placed into a J.L. Shepherd and Associates model 143-45 Cesium-137 irradiator. Mice in each group received a subcutaneous treatment of 0 (vehicle), 5, 10, or 20 μmol $Cu(II)_2(3,5\text{-DIPS})_4$/kg of body mass three hours post-irradiation. Survival at the end of a 30-day period after irradiation was determined as the measure of radiation recovery. Statistical significance of survival data was analyzed by the Fisher Exact Two-Tailed method. Comparisons of body mass measurements and locomotion counts were analyzed using the one-way ANOVA. Post-hoc Comparisons were made using Dunnett's test ($P<0.05$).

### Results

$Cu(II)_2(3,5\text{-DIPS})_4$ did facilitate the increase in survival of irradiated mice. As shown in Figure 1, mice treated with 0, 5, 10, or 20 μmol $Cu(II)_2(3,5\text{-DIPS})_4$/kg of body mass 3 hours after $LD_{0/30}$ irradiation exhibited survivals of 32%, 52%, 60%, or 52% respectively. These data represent increases in survival of 63% for the 5 and 20 μmol/kg of body mass-treated groups and an 88% increase in survival of the 10 μmol/kg of body mass-treated group which is significantly ($P<0.05$) greater than survival observed for vehicle-treated mice. Treatment, as anticipated, did not cause acute toxicity in that irradiated mice treated with various doses of $Cu(II)_2(3,5\text{-DIPS})_4$ did not die before mice treated with vehicle. Mice in the vehicle-treated group began dying before mice in the $Cu(II)_2(3,5\text{-DIPS})_4$-treated groups.

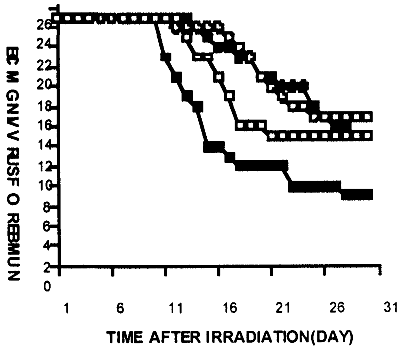

Figure 1. Survival of female C57BL/6 mice treated with vehicle (■), 5 μmol (●), 10 μmol (O), or 20 μmol ( )$Cu(II)_2(3,5\text{-DIPS})_4$/kg of body mass 3 hr after day zero, the day of irradiation.

Average body mass of $Cu(II)_2(3,5\text{-DIPS})_4$-treated mice recovered more rapidly than that of the vehicle-treated control group. Figure 2 shows the average daily body mass for vehicle-treated $Cu(II)_2(3,5\text{-DIPS})_4$-treated groups. The average body mass for all treatment groups followed approximately the same trend until day 14 when the 20 μmol/kg of body mass-treated group began a sharp increase in average body mass which became significantly ($P<0.05$) different from the increase in body mass observed for the vehicle-treated group on day 30. A less dramatic and not statistically significant increase in average body mass was exhibited by the 10 μmol/kg of body mass-treated group on day 21 and remained distinct from both the 0 μmol/kg of body mass and 5 μmol/kg of body mass groups through day 30. The vehicle-treated and the 5 μmol/ kg of body mass-treated groups exhibited similar and not significantly different changes in average body mass throughout the study. Treatment with 10 μmol/kg or 20 μmol/kg appears to have produced a dose-related increase in body mass that was observed after days 21 or 16 respectively.

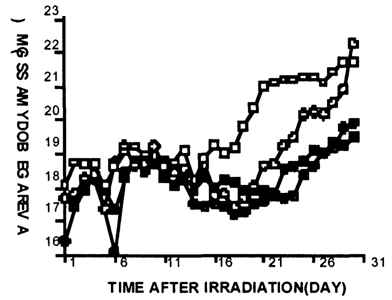

Figure 2. Average body mass change for female C57BL/6 mice treated with vehicle (■), 5 μmol (●), 10 μmol (O), or 20 μmol ( ) $Cu(II)_2(3,5\text{-}DIPS)_4$/kg of body mass 3 hr after day zero, the day of irradiation.

All groups displayed a decrease in average daily locomotion activity following irradiation (Figure 3). $Cu(II)_2(3,5\text{-}DIPS)_4$-treated groups began to increase locomotor activity between days 13 and 15 which continued to increase until day 30. Locomotion of the vehicle-treated group recovered only slightly by day 30. Average daily locomotor activity for all $Cu(II)_2(3,5\text{-}DIPS)_4$-treated mice rapidly recovered from day 15 onward and this recovery was significantly ($P \leq 0.001$) related to increasing dose of $Cu(II)_2(3,5\text{-}DIPS)_4$ on day 30.

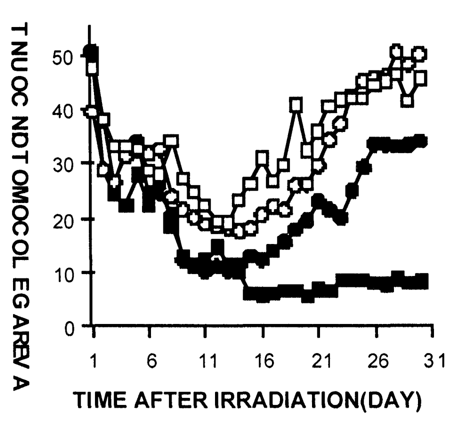

Figure 3. Average change in locomotion of female C57BL/6 mice treated with vehicle (■), 5 μmol (●), 10 μmol (O), or 20 μmol ( ) $Cu(II)_2(3,5\text{-}DIPS)_4$/kg of body mass 3 hr after day zero, the day of irradiation.

### Discussion

The initial phase of Performance Decrement is correlated with a drop in blood pressure and cerebral blood flow [15]. Nitric Oxide (NO), the Vascular Endothelium Derived Relaxing Factor(EDRF) has been shown to be a potent vasodilator and hypotensive agent [15]. Nitric Oxide has also been shown to be found in the largest quantities, with respect to other parts of the brain, in the cerebellum [15]. The possibility that radiation might increase the production of NO would account for the observed decrease in blood pressure and cerebral blood flow [17]. A compound that would down-regulate nitric oxide synthase and thus the production of NO would therefore attenuate the decrease in blood pressure and cerebral blood flow.

Chemicals that directly modulate these two physiological events were studied in order to attenuate behavioral disruption. Direct attenuation with the blood pressure stabilizer norepinephrine was not successful, possibly because the relationship between behavior and blood pressure changes is not always direct [17]. A substance that will prevent the loss of blood flow to cerebral areas and restore normal blood pressure by inhibiting mechanisms responsible for these decrements is needed rather than a substance that simply treats the occurrence of these events.

Recent studies suggest that $Cu(II)_2(3,5\text{-}DIPS)_4$ down-regulates Nitric Oxide Synthase [18] which is necessary for the production of NO. Inhibition of NO synthesis may prevent radiation-induced vasodialation and hypotension. It has also been suggested that the regulation of vasodilation may play a role in radiation protection (18). The present report offers support for the possiblity that recovery from radiation-induced vasodilation plays a role in radiation recovery.

It has been shown that post-irradiation treatment with non-toxic doses of $Cu(II)_2(3,5\text{-}DIPS)_4$ increases the rate of recovery of immunocompetency and histopathology as well as survival of irradiated mice [10,11]. These reports included data showing the Therapeutic Index, $LD_{50}/ED_{50}$, of $Cu(II)_2(3,5\text{-}DIPS)_4$ to be 13.1 when using a 20 μmol/kg body of mass dose, the largest dose used in the present studies, to achieve radioprotection /radiorecovery. It should be noted that 10 is the ideal Therapeutic Index, which is a measure of safety in use.

Treatment with non-toxic doses of $Cu(II)_2(3,5\text{-}DIPS)_4$ used in this study also enabled rapid recovery of radiation-induced impaired locomotion and loss of body mass. Since radiation causes long-term impairment, which is not corrected with existing radioprotectants, the use of $Cu(II)_2(3,5\text{-}DIPS)_4$ offers an additional advantage as a radioprotectant and radiorecovery agent.

### Conclusion

Results obtained in this study strongly support the hypothisis that $Cu(II)_2(3,5\text{-}DIPS)_4$ is effective in facilitating the recovery of radiation-induced behavioral decrements and confirm the previous report that treatment with $Cu(II)_2(3,5\text{-}DIPS)_4$ after $LD_{50/30}$ irradiation is an effective radiorecovery measure. This radiorecovery activity is consistent with rapid recovery of immunocompetency, repair of radiation-induced histopathology, and impaired locomotion.

### Acknowledgements

Support for this research was provided via Arkansas Space Grant Consortium Student Fellowships NL-3013L (T.D.H.) and NL-3013H (R.D.H.) funded by the National Aeronautics and Space Administration and the Arkansas Science and Technology Authority; National Cancer Institute, Summer Student Fellowship (H.J.I.), PHS grant Number CA49425; and the National Institute for General Medical Sciences, PHS grant number S06-RR08211.

### References

[1] D. L. Collins, A. B. De Carvalho, *Behav. Med.* Vol. **18** (1993) 149.
[2] R. I. Walker, *Pharmacol. Ther.* **39** (1988) 13.
[3] D. M. Maier, M. R. Landauer, *Aviat. Space Environ. Med.* **60** (1989) 774.
[4] D. M. Maier, M. R. Landauer *Aviat. Space Environ. Med.*, **61** (1990) 893.
[5] W. F. Burghardt, W. A. Hunt, *Pharmacol. Biochem. Behav.* **33** (1989) 549.
[6] V. Bogo, *Pharmacol Ther.* **39** (1988) 73.
[7] M. R. Landauer, H. D. Davis, J. A. Dominitz, J. F. Weiss, *Toxicology*, **49** (1988) 315.
[8] M. R. Landauer, H.D. Davis, J. A. Dominitz, J. F. Weiss, Abstract Ep-29, Thirty-Eighth Annual Meeting of the Radiation Research Society, April 7-20, 1990.
[9] M. R. Landauer, H. D. Davis, J. A. Q. Dominitz, J. F. Weiss, *Pharmacol. Biochem. Behav.* **27** (1987) 573.
[10] J. R. J. Sorenson, L. S F. Soderberg, M. L. Baker, J. B. Barnett, L. W. Chang, H. Salari, W. M. Willingham, in *"Antioxidants in Therapy and Preventive Medicine"* (I. Emerit, C. Auclair, L. Packer, Eds.) Plenum Press, New York, (1990) 69.
[11] J.R.J. Sorenson, L.S.F. Soderberg, L.W. Chang, *Proc. Soc. Exptl. Biol. Med.* **210** (1995) 191.
[12] F.T. Greenaway, L.J. Norris, J.R.J. Sorenson, *Inorg. Chim. Acta* **145** (1988) 279.
[13] J.J. Reiners, Jr., E. Brott, J.R.J. Sorenson, *Carcinogenesis* **7** (1986) 1729.
[14] J.R.J. Sorenson, E.G. Melby, P.J.Nord, H.G. Petering, *Arch. Environ. Health* **27** (1973) 36.
[15] L.G. Cockerham, B.J. Kelman, *Fund. Appl. Tox.* **11** (1988) 571.
[16] R.A. Johns, J.C. Moscicki, C.A. Difazio, *Anesthe.* **77** (1992) 779.
[17] G. Deliconstantinos, V. Villiotou, C. Fassitsas, *J. Cardiovas. Pharmacol.* **20** (Suppl. 12) (1992) S63.
[18] J.G.L. Baquial, J.R.J. Sorenson, *J. Inorg. Biochem.* **60** (1995) 133.

[19] J.C. Debouzy, Y. Chancerelle, F. Fauvelle, H. Vezin, C. Marroncles, C. Cruz, J.M. Maillet, *Biol. Chim. Farmaceut.* **133** (1994) 228.

**Received: March 2, 1999 - Accepted: March 31, 1999 - Received in revised camera-ready format: April 1, 1999**

